# Mind wandering during first- and foreign-language reading

**DOI:** 10.3758/s13423-026-02949-w

**Published:** 2026-06-30

**Authors:** Marina Klimovich, Jean-François Rouet, Tobias Richter

**Affiliations:** 1 University of Würzburg, Department of Psychology IV, Röntgenring 10, 97070 Würzburg, Germany; 2 Center for Research on Cognition and Learning, University of Poitiers, Poitiers, France

**Keywords:** Mind wandering, Bilingualism, Reading comprehension, Text difficulty

## Abstract

This research investigated whether individual differences in vocabulary knowledge influence mind wandering during first language (L1) and foreign language (L2) reading. In four independent studies involving different L1 and L2 pairings – German and English (Study 1), French and English (Study 2), English and German (Study 3), and German and French (Study 4) – participants completed measures of mind-wandering frequency, perceived reading difficulty, and comprehension. Studies 2–4 additionally assessed the intentionality of mind wandering. Across all studies, participants with lower vocabulary knowledge in their L2 compared to L1 showed higher frequencies of mind wandering during L2 compared to L1 reading. Studies 1 and 2 additionally included participants with comparable L1/L2 vocabulary knowledge and found no significant L1–L2 differences in mind wandering. Consistent with previous research, our findings suggest that the intentionality of mind wandering is sensitive to task demands. When L2 vocabulary knowledge impaired comprehension on the textbase and situation model levels, participants showed increased unintentional but not intentional mind wandering in L2 compared to L1 (Studies 3 and 4). Under these conditions, task demands likely exceeded available executive-control resources, allowing automatic and unintentional task-unrelated thoughts to intrude. In contrast, when L2 vocabulary supported comprehension at least on the textbase level (Studies 1 and 2), metacognitive evaluations became more influential. Specifically, participants showed more intentional but not unintentional mind wandering in L2 compared to L1, and perceived difficulty mediated the relationship between L2 vocabulary knowledge and mind wandering. Here, participants likely disengaged deliberately when the cognitive costs of L2 reading outweighed its perceived benefits.

## Introduction

Effectively comprehending a text requires maintaining focused attention. However, about 30% of the time, readers lose attention and engage in thoughts unrelated to the text, a phenomenon known as *mind wandering* (Wong et al., 2022). Mind wandering during reading has been shown to hinder extracting information from written texts and the ability to make inferences, leading to reduced comprehension performance (Bonifacci et al., 2023; D’Mello & Mills, 2021). Given the detrimental impact of mind wandering on comprehension, understanding conditions that make mind wandering likely is crucial.

In the context of reading, one of the most extensively examined predictors of mind wandering is text difficulty (D’Mello & Mills, 2021). However, the relationship between these variables is complex because mind wandering has been shown to decrease (Fulmer et al., 2015) and increase (Feng et al., 2013; Mills et al., 2015; Soemer & Schiefele, 2019) with text difficulty. For example, in a study by Fulmer et al. (2015), college students previewed summaries of educational texts and ranked them based on personal preference. They then read texts designed to be either easy or difficult for each ranked topic. During reading, participants were intermittently asked to indicate whether their mind was wandering. The data revealed an interaction effect between text difficulty and rank order, indicating that mind wandering was more prevalent when participants read easy texts on less-preferred topics compared to difficult texts. This finding aligns with the executive resource hypothesis, which suggests that a primary activity (e.g., reading) and mind wandering compete for limited cognitive resources (Smallwood & Schooler, 2006). Easier texts require less cognitive control, thereby allowing more resources to be allocated to mind wandering. This account is further supported by numerous studies in other domains such as visual or auditory perception or working memory (e.g., Forster & Lavie, 2009; McKiernan et al., 2006).

Contrary to the executive resource hypothesis, several studies suggest that difficult texts are more prone to inducing mind wandering (Feng et al. 2013; Kahmann et al., 2022; Mills et al., 2015; Soemer & Schiefele, 2019). For example, Feng et al. (2013) found that participants experienced more frequent mind wandering when reading the difficult passages from a standardized reading comprehension test than when reading easy passages. The authors attributed this finding to readers encountering more difficulty constructing a situation model when reading difficult texts. A study by Kahmann et al. (2022) supports this interpretation. In their study, participants read texts that varied along five levels of difficulty. Despite testing the hypothesis that mind wandering would follow a U-shaped pattern with text difficulty (i.e., with peaks during very easy and very difficult texts compared to moderately difficult texts), they found a linear increase in mind wandering with greater text difficulty. The executive control failure account (Kane & McVay, 2012; McVay & Kane, 2012) explains the increase in mind wandering during difficult tasks by suggesting that mind wandering is initiated when executive control fails in effectively performing a primary task, allowing alternative thoughts to enter the focus of attention.

Research on text difficulty manipulation has provided insights into how processing demands affect mind wandering, but language proficiency has been largely overlooked as one potentially critical variable. Reading in a language with low proficiency requires more cognitive resources to maintain sustained attention on comprehension than reading in a language with high proficiency (Morishima, 2013). Readers need to process surface-level information (e.g., word decoding) while simultaneously upholding a coherent mental model of the text (van Dijk & Kintsch, 1983; Zwaan & Radvansky, 1998). This dual cognitive demand often leads to lower overall comprehension in second-language reading (Morishima, 2013; Zwaan & Brown, 1996). Since prominent accounts on mind wandering indicate that task demands are closely linked to attention, reading a text that does not match a person’s language proficiency should impact the frequency of mind wandering.

The present study aimed to extend our understanding of the relationship between the difficulty of processing text and mind-wandering frequency by investigating individual differences in participants’ proficiency in their first (L1) and foreign (L2) language. During skilled reading, such as in L1, lower-level linguistic processes that are involved in forming the textbase are largely automatic and demand minimal cognitive resources (Perfetti, 2007). The routinization of lower-level reading processes in L1 enables readers to allocate cognitive resources to higher-level processes, such as integration and inference making, which facilitate and improve discourse comprehension (Perfetti, 2007; Zwaan & Brown, 1996). In contrast, when reading in a less proficient language, the lower-level linguistic processes that are needed to construct the textbase require more cognitive resources. L2 readers often rely on translating words from L2 to L1 to comprehend their meaning, rather than directly associating L2 words with their conceptual representations in the mental lexicon (Morishima, 2013; Zwaan & Brown, 1996). Consequently, the increased cognitive load on lower-level processes diminishes the resources available for higher-level activities and thus impairs the ability to form a coherent situation model and achieve comprehension (Zwaan & Brown, 1996). We propose that the greater cognitive demands of reading in L2 and the associated difficulties in situation model construction will lead to more executive control failures and thus a higher mind-wandering frequency.

In addition to actual text difficulty, perceptions of difficulty might affect subjective evaluations and consequently also influence mind wandering. For example, Forrin et al. (2019) demonstrated that readers perceived texts with longer sections as being more difficult, despite identical content, and exhibited increased mind wandering (i.e., section-length effect). The authors concluded that the perception of increased difficulty might have caused participants to disengage from texts with longer sections. According to the cost-benefit account (Kurzban et al., 2013; Shenhav et al. 2017), participants associate tasks they perceive as cognitively demanding with high opportunity costs, which results in task disengagement. To test the assumption whether processing difficulty (actual or perceived) drives the effect of L2 language proficiency on mind wandering, we collected participants’ ratings of reading difficulty.

Furthermore, research has emphasized the importance of differentiating between intentional and unintentional mind wandering (Barrington & Miller, 2023; Forrin et al., 2021; Seli et al., 2016). Unintentional mind wandering describes episodes in which attention drifts from the reading task without deliberate disengagement, whereas intentional mind wandering involves a deliberate decision to think about something unrelated to the text. In non-reading contexts, research suggests that intentional mind wandering is more frequent during less demanding tasks, whereas unintentional mind wandering is more frequent during cognitively demanding tasks and is often associated with lower working-memory capacity (Barrington & Miller, 2023; Ju & Lien, 2018; Robison & Unsworth, 2018). In reading, however, Soemer and Schiefele (2019) found that higher text difficulty increased intentional and unintentional mind wandering, and in later work they found negative correlations between types of mind wandering and working memory capacity (Soemer & Schiefele, 2020). Regarding perceived difficulty, Forrin et al. (2021) showed that readers were more likely to mind wander unintentionally when reading long compared to short text sections. To gain a clearer understanding of the mechanisms through which language proficiency might influence mind wandering, the present research distinguished between intentional and unintentional mind wandering.

To assess participants’ proficiency in L1 and L2, we administered a vocabulary test alongside a self-report measure. Reading comprehension is closely tied to word knowledge and the quality of the corresponding lexical representations (Perfetti, 2007). High language exposure and thus higher vocabulary knowledge are linked to stronger representations of words and more efficient lexical access (Diependaele et al., 2013; Yap et al., 2012). This ease of word processing enables readers with high vocabulary knowledge to make more inferences and achieve higher comprehension (Currie & Cain, 2015; Jeon & Yamashita, 2014). Considering the importance of vocabulary knowledge in processing demands and comprehension, a vocabulary test provides a valid and economic proxy of language proficiency.

### The present research

The main aim of the present research was to examine whether language proficiency affects mind wandering during reading. Prior work on the relationship between processing demands and mind wandering has yielded mixed findings, with studies reporting increased mind wandering for easier and more difficult texts, as well as non-linear relationships depending on the task. To address this inconsistency, we adopted a complementary approach by focusing on individual differences in language proficiency rather than manipulating text characteristics. According to the executive-control failure account, mind wandering occurs when executive control fails to maintain focus on the primary task, allowing task-unrelated thoughts to arise. Higher task demands increase the likelihood of such failures. Reading in a less proficient language (i.e., with lower vocabulary knowledge) is typically more demanding than reading in a more proficient language and can hinder the construction of a situation model.

We conducted four studies with identical designs but sampled from different populations. We measured mind-wandering frequency among participants with varying levels of L1/L2 vocabulary knowledge as they read texts in their L1 German/L2 English (Study 1), L1 French/L2 English (Study 2), L1 English/L2 German (Study 3), and L1 German/L2 French (Study 4). Using the same materials across four different populations provided robustness to our findings and allowed us to examine their generalizability. We expected that participants with unequal L1/L2 vocabulary knowledge would mind-wander more frequently in L2 compared to L1 (Hypothesis 1). Furthermore, we conducted mediation analyses to test the assumption that increased reading difficulty accounts for the effect of L2 vocabulary knowledge on mind wandering (Hypothesis 2).

To gain insight into the processes underlying the relationship between processing difficulty and mind wandering, Studies 2–4 distinguished between intentional and unintentional mind wandering. One possibility is that the increased cognitive load during L2 reading exceeds available executive-control resources (Kane & McVay, 2012; McVay & Kane, 2012), making it more difficult to maintain task focus and thereby allowing task-unrelated thoughts to intrude (i.e., unintentional mind wandering). Alternatively, (perceived) effortful L2 reading may cause the expected benefits of continued engagement to no longer outweigh the associated cognitive costs (Kurzban et al., 2013; Shenhav et al., 2017), prompting readers to intentionally disengage. We adopted an exploratory approach to examine whether participants with unequal L1/L2 vocabulary knowledge would exhibit increased intentional mind wandering (Exploratory Analysis 1), unintentional mind wandering (Exploratory Analysis 2), or both during L2 reading. All studies were preregistered except Study 1 (https://aspredicted.org/wz7bt9.pdf).

## Method

### Design and power

To test our hypotheses, we used a 2 (between: participants with equal L1/L2 vocabulary knowledge vs. unequal L1/L2 vocabulary knowledge) × 2 (within: L1 vs. L2) mixed design. Participants with the following language backgrounds took part in our studies: L1 German versus L2 English (Study 1), L1 French versus L2 English (Study 2),^1^ L1 English versus L2 German (Study 3), and L1 German versus L2 French (Study 4). Assuming a medium effect (*f* =.25), a sample of 54 students is required to detect the interaction of vocabulary balance and language at an α-level of.05 and a power (1 – β) of .95, assuming medium correlations (ρ =.5) between repeated measures (computed with the G*Power 3 software; Faul et al., 2007). We deliberately targeted a sample size of 70 participants to account for data loss.

Language background and vocabulary knowledge were assessed with a language history questionnaire based on the Language Experience and Proficiency Questionnaire (LEAP-Q; Marian et al., 2007), and the lexical decision based LexTale in English, German (Lemhöfer & Broersma, 2012), and French (Brysbaert, 2013). Participants were classified as having comparable L1/L2 vocabulary knowledge when their L2 LexTale score reached 80% or above, signifying proficiency levels C1 and C2 within the Common European Framework of Reference for Languages (Lemhöfer & Broersma, 2012). Additionally, participants with a difference of less than 10% between their L1 and L2 LexTale scores were also regarded as having comparable L1/L2 vocabulary knowledge. The latter criterion ensured that participants with generally low but comparable vocabulary scores (e.g., L1 = 50%, L2 = 46%) were not erroneously categorized as having unequal vocabulary knowledge. Table 1 provides an overview of the participants’ self-reported and objective language characteristics. The local ethics board granted ethical approval for all studies (Studies 1 and 3: GZEK 2022-72, Studies 2 and 4: GZEK 2025-23).

**Table 1:** Participants’ language background and vocabulary knowledge

Studies	Study 1				Study 2				Study 3				Study 4			
	Comparable L1/L2 (*n* = 31)		Unequal L1/L2 (*n* = 41)		Comparable L1/L2 (*n* = 42)		Unequal L1/L2 (*n* = 30)		Comparable L1/L2 (*n* = 9)		Unequal L1/L2 (*n* = 61)		Comparable L1/L2 (*n* = 4)		Unequal L1/L2 (*n* = 66)	
	L1 German	L2 English	L1 German	L2 English	L1 French	L2 English	L1 French	L2 English	L1 English	L2 German	L1 English	L2 German	L1 German	L2 French	L1 German	L2 French
Self-report																
AoA L2 acquisition	-	8.42 (1.88)	-	8.44 (2.06)	-	9.15 (2.89)	-	10.00 (2.85)	-	-	-	9.36 (5.31)	-	-	-	11.23 (3.69)
Age started L2 reading	-	10.30 (2.33)	-	9.63 (2.03)	-	12.49 (5.04)	-	14.68 (7.76)	-	-	-	10.95 (4.25)	-	-	-	11.98 (3.66)
Extent of exposure^a^	5.13 (1.66)	4.15 (1.44)	6.46 (1.56)	3.73 (1.60)	7.34 (1.60)	4.57 (1.82)	8.21 (1.52)	3.59 (1.43)	-	-	7.96 (1.33)	3.03 (2.02)	-	-	6.46 (1.83)	2.06 (1.87)
Proficiency^a^	9.24 (0.75)	7.97 (0.96)	9.16 (0.67)	6.93 (1.62)	9.50 (0.63)	7.50 (1.94)	9.57 (0.64)	6.23 (2.59)	-	-	9.80 (0.40)	7.33 (1.20)	-	-	9.47 (0.75)	5.98 (2.19)
Performance																
LexTale Accuracy (%)	89.19 (5.47)	84.56 (7.51)	85.79 (5.27)	64.88 (7.40)	84.08 (8.38)	81.79 (7.99)	86.31 (8.12)	70.50 (7.19)	-	-	92.19 (6.93)	62.41 (9.16)	-	-	85.33 (10.21)	52.33 (8.61)

AoA = age of acquisition. Standard deviations are displayed in brackets

^a^ Range 0 (low) to 10 (high)

### Participants

Studies 1 and 4 were conducted with participants from the participant database of the University of Würzburg. Studies 2 and 3 were conducted using the crowdsourcing platforms Clickworker and Prolific, respectively. Participants were excluded from analyses if they neglected to report the corresponding L1 relevant to the specific study. For the online studies (Studies 2 and 3), we additionally implemented the following exclusion criteria: Participants were excluded if they failed instructed response questions, which served as an attention check (Kennedy et al., 2020), or they provided inconsistent information between their birth year at the start of the study and their age later in the study (Chmielewski & Kucker, 2020). Although not preregistered, we additionally verified whether participants read a text at a rate exceeding 650 words per minute – a threshold typically associated with skimming or scanning rather than normal reading (Brysbaert, 2019; Carver, 1992). The imbalance in sample size between comparable and unequal L1/L2 vocabulary groups in Studies 3 and 4 reflects discrepancies between self-reported and objectively assessed proficiency. Participants were recruited based on self-report, and group assignment was determined post hoc using the vocabulary measure LexTale. Pre-screening was not implemented for ethical and practical reasons because it would have required excluding participants after recruitment.

We recruited 77 participants for Study 1. Four participants were excluded from analyses because they reported an L1 other than German, and one participant was excluded because of a technical error that resulted in a loss of data during the study. The final sample therefore comprised *N* = 72 (60 women, 12 men, 0 other). On average, participants were 24.38 years old (*SD* = 7.67 years). Based on the results of the LexTale, participants were categorized into having comparable L1/L2 vocabulary knowledge (*n* = 31) or unequal L1/L2 vocabulary knowledge (*n* = 41).

We recruited 88 participants for Study 2. Participants were excluded from analyses when they did not report French as their L1 (*n* = 3), when they failed the attention checks (*n* = 2), and when they read a text at a rate exceeding 650 words per minute (*n* = 11). The final sample therefore comprised *N* = 72 (35 women, 37 men, 0 other; *M*_age_ = 33.58 years, *SD*_age_ = 7.88 years). Based on the results of the LexTale, participants were categorized into having comparable L1/L2 vocabulary knowledge (*n* = 42) or unequal L1/L2 vocabulary knowledge (*n* = 30).

We recruited 76 participants for Study 3. One participant was excluded for not reporting English as their L1. No participants failed the attention checks. Five participants were excluded for reading at a rate exceeding 650 words per minute. Based on the results of the LexTale, participants were categorized into having comparable L1/L2 vocabulary knowledge (*n* = 9) or unequal L1/L2 vocabulary knowledge (*n* = 61). Given the small number of participants with comparable L1/L2 vocabulary knowledge, analyses were restricted to the subgroup with unequal L1/L2 vocabulary knowledge. The final sample therefore consisted of 61 participants (27 men, 29 women, five other; *M*_age_ = 28.49 years, *SD*_age_ = 5.50 years).

We recruited 74 participants for Study 4. Four participants were excluded from analyses because they reported an L1 other than German. Based on the results of the LexTale, participants were categorized into having comparable L1/L2 vocabulary knowledge (*n* = 4) or unequal L1/L2 vocabulary knowledge (*n* = 66). Given the small number of participants with comparable L1/L2 vocabulary knowledge, analyses were restricted to the subgroup with unequal L1/L2 vocabulary knowledge. The final sample therefore consisted of 66 participants (13 men, 52 women, 0 other, one missing value; *M*_age_ = 29.11 years, *SD*_age_ = 9.61 years).

### Materials

The text materials were identical in all studies. They consisted of two texts, one discussing the efficacy of psychoanalysis and cognitive behavioral therapy (therapy text, adapted from Maier et al., 2018) and the other discussing whether German should be promoted as a language of science dissemination (science text, Klimovich & Richter, 2024). The comparability between the texts and the target sentences was ensured by holding word length and word frequency strictly parallel. The translations of the texts resulted in slightly different word counts between German (therapy text: 1,075 words, science text: 1,043 words), French (therapy text: 1,075 words, science text: 1,043 words), and English (therapy text: 1,140 words, science text: 1,050 words). Readability scores were determined with the Flesch Reading Ease Index (Flesch, 1948) for the English texts (therapy text: 30, science text: 34), with the German adaptation of the Flesch Reading Ease Index (Amstad, 1978) for the German texts (therapy text: 29, science text: 33), and with the French adaptation of the Flesch Reading Ease Index (Kandel & Moles, 1958) for the French texts (therapy text: 33, science text: 33).

Participants read the texts one sentence at a time, with only a single sentence visible on the screen at any given moment (i.e., self-paced moving window method). Mind-wandering probes were triggered when participants advanced beyond predetermined target sentences in the text, therefore probes were tied to specific locations in the text rather than to fixed time intervals (e.g., Smallwood et al., 2008). The target sentences, along with the mind-wandering probes, occurred on average every 215.40 words after the preceding probe (ranging from 154 to 267 words). When the probe occurred, participants were asked to report whether they were reading the last sentence or thinking about something else. In Studies 2–4, we additionally assessed the intentionality of mind wandering. Whenever participants reported mind wandering, they were further asked to indicate whether it had been intentional or unintentional. After answering the question, participants resumed reading the text sentence by sentence. In total, participants responded to the mind-wandering probe four times per reading in both L1 and L2.

### Procedure

The procedure was identical in all studies, except that Studies 1 and 4 were conducted in a laboratory setting and Studies 2 and 3 were conducted online via crowdsourcing platforms. All studies were conducted using Inquisit. First, participants’ working memory capacity (reading span; German: Oberauer et al., 2000; English: Conway et al., 2005; French: Gonthier et al., 2016) and prior knowledge of the text topic (i.e., five multiple-choice questions per text) were assessed. Prior to reading the target texts, participants were given a short sample text to familiarize them with the sentence-by-sentence self-paced reading procedure and mind-wandering probes. They were then presented with one text in their L1 and one in L2. The texts were counterbalanced across participants and languages. They either read the therapy text first or the science text first, and they either began with the text presented in their L1 or the text presented in their L2.

After reading each text, participants provided metacognitive judgments about their reading experience. They rated their perceived difficulty of the text on three items, each on a 7-point scale (0 = “strongly disagree”; 6 = “strongly agree”): “It was difficult for me to read the text”; “I often had to read sentences multiple times to understand their meaning”; and “I found the language used in the text challenging.” Afterwards, they rated their interest in the topic (scale adapted from Soemer & Schiefele, 2019) and motivation (scale adapted from Unsworth & McMillan, 2013).

Following these metacognitive judgments, participants’ comprehension performance was assessed at the levels of the textbase and situation model following the procedure developed by Schmalhofer and Glavanov (1986). Participants responded to three types of items: paraphrases, inferences, and distractors. Paraphrase items rephrased information explicitly stated in the text (e.g., by altering word order or replacing key words with synonyms), whereas inference items contained information that could be inferred from the text but was not explicitly stated. Distractor items included information that was neither explicitly mentioned nor inferrable from the text. In total, participants responded to 24 test items per text (eight items per item type). The items were presented in the same language in which participants read the texts. The textbase score was obtained by subtracting the probit-transformed proportion of “yes” responses to distractor items (false alarms) from the probit-transformed proportion of “yes” responses to paraphrase items (hits). The situation model score was obtained by subtracting the probit-transformed proportions of “yes” responses to the distracter items (false alarms) from the probit-transformed proportions of “yes” responses to the inference items (hits). Given that a probit transform returns a *z-*value representing the corresponding point on the cumulative normal distribution, we added a constant of 5 to avoid negative values in the dataset (e.g., Cohen et al., 2003, p. 241; Maier & Richter, 2013). Examples of the test items are available on the website of the Open Science Framework (OSF) (https://osf.io/4tx2s/overview?view_only=ccec5d5b0eae4b9b977be8d1a56b5aa8).

Finally, participants’ L1 and L2 language proficiency and trait-based mind wandering were assessed (Mrazek et al., 2013).

### Data analysis strategy

The analyses were conducted in accordance with preregistration, unless stated otherwise (https://aspredicted.org/wz7bt9.pdf). Three generalized mixed-effects models with a logit link were constructed for (1) overall mind wandering (the combination of intentional and unintentional mind-wandering responses), (2) intentional mind wandering, and (3) unintentional mind wandering (note that Study 1 examined only overall mind wandering). Two linear mixed-effects models were constructed for comprehension performance on the textbase and situation model level. Utilizing mixed-effects models enables analyses at the subject and sentence level, avoiding the need to average data across participants or sentences, which could otherwise result in misleading conclusions (Baayen et al., 2008; Linck & Cunnings, 2015). Vocabulary balance (0 = participants with comparable L1/L2 proficiency, 1 = unequal L1/L2 vocabulary knowledge), language (0 = L1, 1 = L2), and their interaction were included as fixed effects (dummy-coded) in all models.

All four studies followed the same analytical approach, with one exception in Studies 3 and 4. We restricted the analyses to participants with unequal L1/L2 vocabulary knowledge because we were unable to recruit sufficient participants with comparable L1/L2 vocabulary knowledge. Therefore, the models for Studies 3 and 4 included only the main effect of language. The main effect of vocabulary balance and the interaction between vocabulary balance and language were omitted. Given that our hypotheses concerned differences in mind wandering between L1 and L2 specifically within the subgroup with unequal L1/L2 vocabulary knowledge, all preregistered hypotheses could still be tested.

We employed the maximal random-effects structure based on the design of our studies (Barr et al., 2013). In the models for comprehension performance, participants were included as random effects. In the model for mind-wandering probability, participants and sentences were included with crossed random intercepts. Additionally, we included by-subject random slopes for the within-subjects fixed-effect language to account for random variation in the repeated measure (Barr et al., 2013; Linck & Cunnings, 2015). To address convergence issues, we used the “bobyqa” optimizer. When a model still failed to converge, we simplified it stepwise by removing the random effect that contributed the least amount of variance until convergence was achieved. The models for mind-wandering probability also included *z*-standardized topic interest, working memory, motivation, and prior knowledge as covariates. For ease of interpretation, values were back-transformed from the logit scale and reported as probabilities.

Mediation analyses were conducted to examine whether the effect of L2 vocabulary knowledge on mind wandering can be explained by participants’ perception of increased reading difficulty. Estimates, 95% confidence intervals, and *p-*values for the total, mediated, and direct effect were estimated using a quasi-Bayesian Monte Carlo method with 10,000 simulations. Two models were specified to assess the average causal effects. One model predicts the mediator (perceived difficulty) with L2 vocabulary knowledge as the independent variable, and the other model predicts the outcome variable (mind wandering) with L2 vocabulary knowledge and perceived difficulty as independent variables.

For the generalized mixed models with a binary outcome, effect sizes are reported as odds ratios (*OR*s). For the linear mixed models, effect sizes were estimated using the *t*back approach (Correll et al., 2022), which derives eta squared (η^2^) from the *t* statistic and its associated degrees of freedom to quantify the proportion of variance explained by the effect of interest. To enhance interpretability, we then transformed η^2^ into Cohen’s *d*.

Significance tests were based on α =.05 (one-tailed tests for directed hypotheses). Data were analyzed using R, version 4.4.1 (R Core Team, 2022), and the packages lme4 (Bates et al., 2015), lmerTest (Kuznetsova et al., 2017), mediation (Tingley et al., 2014), emmeans (Lenth, 2016), and ggplot2 (Wickham, 2016). The dataset, the descriptive statistics, the reproducible R code, and the full model results can be found in the OSF repository at: https://osf.io/4tx2s/overview?view_only=ccec5d5b0eae4b9b977be8d1a56b5aa8

## Results

### Mind wandering during L1 and L2 reading

The model estimates and significance tests are shown in Table 2.

**Table 2:** Model results for overall mind wandering, intentional mind wandering, and unintentional mind wandering for Studies 1–4

	Study 1 (L1: German, L2: English)				Study 2 (L1: French, L2: English)				Study 3 (L1: English, L2: German)				Study 4 (L1: German, L2: French)			
	*b*	*SE*	*z*	*p*	*b*	*SE*	*z*	*p*	*b*	*SE*	*z*	*p*	*b*	*SE*	*z*	*p*
Overall mind wandering																
Intercept	−0.81	0.20	−3.99	<.001	−1.06	0.26	−4.16	<.001	−0.95	0.16	−6.05	<.001	−1.30	0.21	−6.33	<.001
Balance	0.20	0.31	0.64	.522	0.02	0.33	0.07	.941	-	-	-	-	-	-	-	-
Language	0.78	0.26	2.96	.003	0.61	0.30	2.02	.044	0.61	0.19	3.26	.001	0.87	0.21	4.15	<.001
Balance × language	−0.78	0.41	−1.92	.028	−0.74	0.40	−1.87	.031	-	-	-	-	-	-	-	-
Random effects	*SD*				*SD*				*SD*				*SD*			
Subject (Intercept)	0.56				0.66				0.57				1.05			
Language (Slope)	0.56															
Intentional mind wandering																
Intercept	-	-	-	-	−2.56	0.38	−6.74	<.001	−3.49	0.41	−8.59	<.001	−3.26	0.57	−5.75	<.001
Balance	-	-	-	-	0.03	0.48	0.06	.951	-	-	-	-	-	-	-	-
Language	-	-	-	-	0.77	0.42	1.83	.068	0.56	0.36	1.55	.121	0.57	0.66	0.86	.387
Balance × language	-	-	-	-	−1.26	0.60	−2.10	.036	-	-	-	-	-	-	-	-
Random effects					*SD*				*SD*				*SD*			
Subject (Intercept)					0.59				1.09				1.46			
Language (Slope)													0.66			
Unintentional mind wandering																
Intercept	-	-	-	-	−1.53	0.28	−5.49	<.001	−1.24	0.16	−7.73	<.001	−1.70	0.20	−8.72	<.001
Balance	-	-	-	-	0.00	0.34	0.01	.995	-	-	-	-	-	-	-	-
Language	-	-	-	-	0.16	0.39	0.40	.690	0.50	0.20	2.54	.011	0.70	0.22	3.21	.001
Balance × language	-	-	-	-	−0.09	0.48	−0.19	.851	-	-	-	-	-	-	-	-
Random effects					*SD*				*SD*				*SD*			
Subject (Intercept)					0.49				0.46				0.71			
Language (Slope)					0.82											

Balance = comparable L1/L2 vocabulary knowledge vs. unequal L1/L2 vocabulary knowledge. Language = L1 vs. L2

To enhance the clarity of presentation, the *z*-standardized control variables (interest, motivation, prior knowledge, and working memory) have been omitted from the table

#### Overall mind wandering

**Fig. 1:**
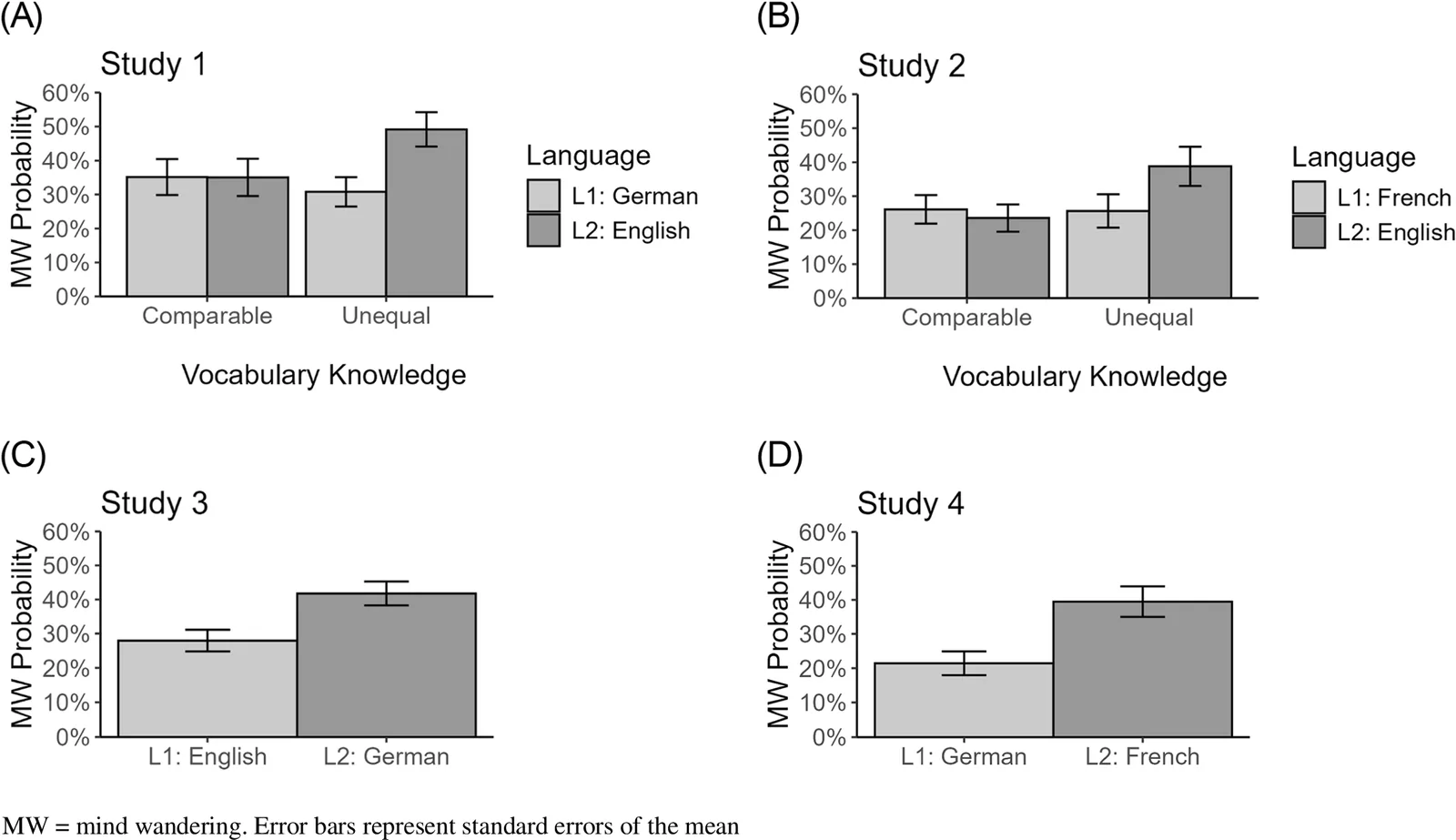
Overall mind-wandering probability in Studies 1**–**4

In Study 1, the interaction between vocabulary balance and language was significant. Participants with unequal L1/L2 vocabulary knowledge showed significantly more mind wandering in L2 compared to L1, *b* = 0.78, *SE* = 0.26,* z* = 2.96, *p* = .001, *OR* = 2.17, whereas no significant difference was found in participants with comparable L1/L2 vocabulary knowledge, *b* = −0.00, *SE* = 0.31, *z* = −0.02,* p* = .988, *OR* = 1.00. In Study 2, the interaction between vocabulary balance and language was significant. Participants with unequal L1/L2 vocabulary knowledge showed significantly more mind wandering in L2 compared to L1, *b* = 0.61, *SE* = 0.30, *z* = 2.02, *p* = .022, *OR* = 1.84, whereas no significant difference was found in participants with comparable L1/L2 vocabulary knowledge, *b* = −0.14, *SE* = 0.27, *z* = −0.52, *p* = .605, *OR* = 0.87. In Studies 3 (*OR* = 1.85) and 4 (*OR* = 2.39), the main effect of language was significant, indicating more mind wandering in L2 compared to L1 among participants with unequal L1/L2 vocabulary knowledge. Therefore, Hypothesis 1 was supported (Fig. 1).

#### Intentional mind wandering

**Fig. 2:**
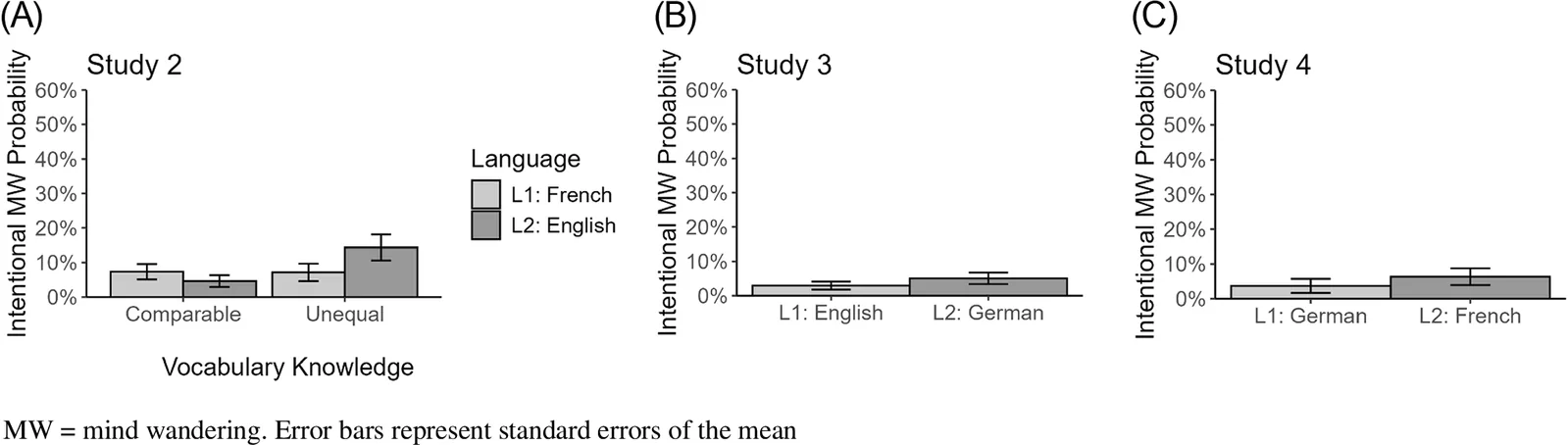
Intentional mind-wandering behavior in Studies 1**–**4

In Study 2, the interaction between vocabulary balance and language was significant. Participants with unequal L1/L2 vocabulary knowledge showed significantly more intentional mind wandering in L2 compared to L1, *b* = 0.78, *SE* = 0.42, *z* = 1.83, *p* = .035, *OR* = 2.17, whereas no significant difference was found in participants with comparable L1/L2 vocabulary knowledge, *b* = −0.49, *SE* = 0.44, *z* = −1.11, *p* = .266, *OR* = 0.61. In Studies 3 (*OR* = 1.75) and 4 (*OR* = 1.76), the main effect of language was not significant. Therefore, the results of Explorative Analysis 1 were mixed (Fig. 2).

#### Unintentional mind wandering

**Fig. 3:**
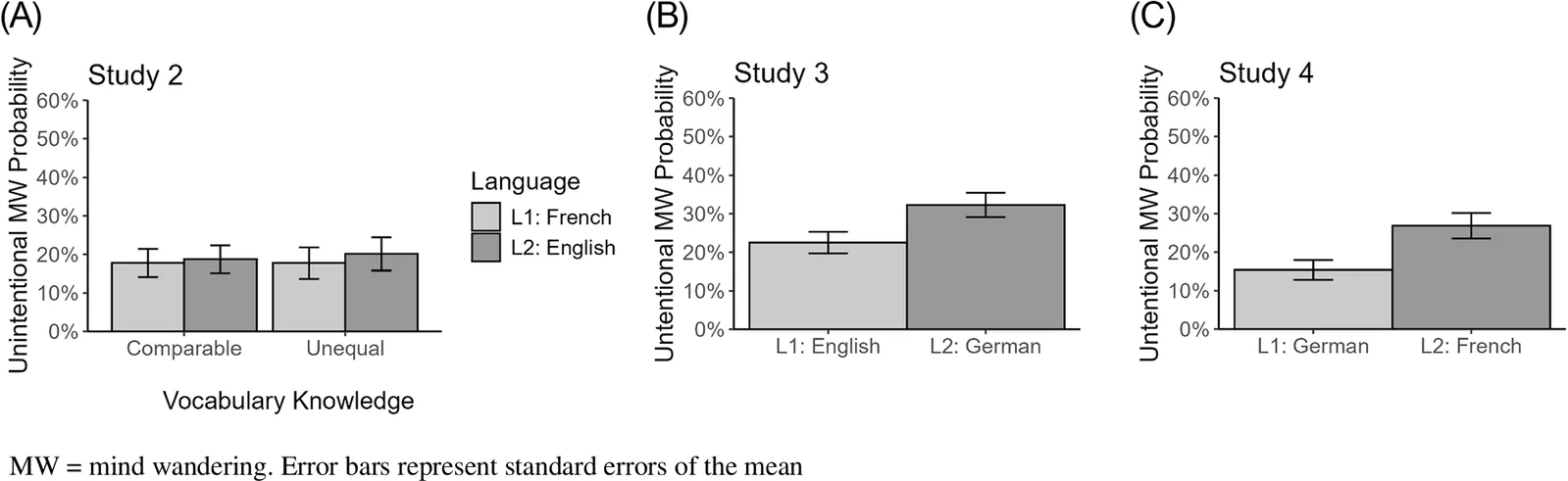
Unintentional mind-wandering behavior in Studies 1**–**4

In Study 2, the interaction effect between vocabulary balance and language was not significant. However, in Studies 3 (*OR* = 1.64) and 4 (*OR* = 2.02), the main effect of language was significant, indicating significantly more unintentional mind wandering in L2 compared to L1 among participants with unequal L1/L2 vocabulary knowledge. Therefore, the results of Explorative Analysis 2 were mixed (Fig. 3).

### Perceived reading difficulty as a mediator for the effect of L2 vocabulary knowledge on mind wandering

Table 3 provides a summary of the estimates, confidence intervals, and *p-*values. Study 1 showed a significant total effect. Participants with lower vocabulary knowledge had a higher mind-wandering frequency. The indirect effect of vocabulary knowledge on mind wandering via perceived reading difficulty was significant, and the direct effect of vocabulary knowledge was also significant. Thus, the effect of L2 vocabulary knowledge on mind wandering was partly mediated by a heightened perception of reading difficulty. Study 2 also showed a significant total effect and indirect effect. The direct effect was not significant, indicating that the effect of L2 vocabulary knowledge on mind wandering was fully mediated by perceived reading difficulty. In contrast, Studies 3 and 4 showed no significant total, direct, or indirect effects. Therefore, Hypothesis 2 was supported by Studies 1 and 2 but not by Studies 3 and 4.

**Table 3:** Perceived reading difficulty as a mediator for the effect of L2 vocabulary knowledge on mind wandering

	Study 1			Study 2			Study 3			Study 4		
Effect	*Est*	95% CI	*p*	*Est*	95% CI	*p*	*Est*	95% CI	*p*	*Est*	95% CI	*p*
Mediation	−0.16	−0.33; −0.03	.010	−0.25	−0.47; −0.07	.002	−0.02	−0.15; 0.10	.750	−0.15	−0.49; 0.12	.280
Direct	−0.29	−0.53; −0.04	.025	−0.24	−0.51; 0.03	.082	0.04	−0.32; 0.40	.810	−0.33	−0.95; 0.30	.300
Total	−0.45	−0.71; −0.18	.001	−0.49	−0.76; −0.22	.001	0.03	−0.35; 0.41	.890	−0.48	−1.10; 0.14	.130

95% CI = 95% confidence interval; Est = Estimate

### Comprehension during L1 and L2 reading

The model estimates and significance tests are shown in Table 4.

**Table 4:** Model results for comprehension on the textbase and situation model level for Studies 1–4

	Study 1 (L1: German, L2: English)					Study 2 (L1: French, L2: English)					Study 3 (L1: English, L2: German)					Study 4 (L1: German, L2: French)				
	*b*	*SE*	*t*	*df*	*p*	*b*	*SE*	*t*	*df*	*p*	*b*	*SE*	*t*	*df*	*p*	*b*	*SE*	*t*	*df*	*p*
Textbase																				
Intercept	1.38	0.13	10.56	125.40	<.001	1.25	0.14	8.76	135.73	<.001	1.00	0.11	9.19	113.57	<.001	1.48	0.12	12.64	117.22	<.001
Balance	0.14	0.20	0.71	125.40	.481	0.04	0.19	0.20	135.73	.840	-	-	-	-	-	-	-	-	-	-
Language	−0.26	0.15	−1.76	70	.083	−0.29	0.18	−1.60	70	.115	−0.28	0.11	−2.48	69	.016	−0.42	0.13	−3.32	69	.001
Balance × language	0.29	0.23	1.29	70	.203	0.11	0.24	0.46	70	.647	-	-	-	-	-	-	-	-	-	-
Random effects	*SD*					*SD*					*SD*					*SD*				
Subject	0.49					0.33					0.62					0.64				
Situation model																				
Intercept	1.55	0.15	10.73	122.60	<.001	1.04	0.16	6.45	132.11	<.001	0.83	0.12	6.98	106.12	<.001	1.36	0.11	11.93	123.64	<.001
Balance	−0.20	0.22	−0.91	122.60	.365	0.08	0.21	0.37	132.11	.716	-	-	-	-	-	-	-	-	-	-
Language	−0.68	0.16	−4.23	70	<.001	−0.38	0.20	−1.94	70	.056	−0.27	0.11	−2.45	69	.017	−0.35	0.13	−2.71	69	.008
Balance × language	0.95	0.25	3.87	70	<.001	0.29	0.26	1.13	70	.263	-	-	-	-	-	-	-	-	-	-
Random effects	*SD*					*SD*					*SD*					*SD*				
Subject	0.57					0.44					0.73					0.56				

Balance = comparable L1/L2 vocabulary knowledge vs. unequal L1/L2 vocabulary knowledge. Language = L1 vs. L2

To enhance the clarity of presentation, the *z*-standardized control variables (interest, motivation, prior knowledge, and working memory) have been omitted from the table

#### Textbase

In Study 1, the interaction effect between vocabulary balance and language was not significant (unequal vocabulary knowledge: L1 *M* = 1.38, *SE* = 0.13, L2 *M* = 1.12*, SE* = 0.13; equal vocabulary knowledge L1 *M* = 1.52, *SE* = 0.15, L2 *M* = 1.55*, SE* = 0.15). In Study 2, the interaction effect between vocabulary balance and language was also not significant (unequal vocabulary knowledge: L1 *M* = 1.25, *SE* = 0.14, L2 *M* = 0.96*, SE* = 0.14; equal vocabulary knowledge L1 *M* = 1.29, *SE* = 0.12; L2 *M* = 1.10*, SE* = 0.12). In Study 3, the main effect of language was significant (*d* = 0.59), indicating lower comprehension on the textbase level in L2 (*M* = 0.72*, SE* = 0.11) compared to L1 (*M* = 0.99, *SE* = 0.11) among participants with unequal L1/L2 vocabulary knowledge. In Study 4, the main effect of language was also significant (L1 *M* = 1.48, *SE* = 0.12, L2 *M* = 1.06*, SE* = 0.12; *d* = 0.80).

#### Situation model

In Study 1, the interaction effect between vocabulary balance and language was significant (*d* = 0.94). Participants with unequal L1/L2 vocabulary knowledge showed a significantly lower comprehension on the situation model level in L2 (*M* = 0.87, *SE* = 0.15) compared to L1 (*M* = 1.56, *SE* = 0.15), *b* = 0.68, *SE* = 0.16, *t*(70) = 4.23, *p* < .001, whereas no significant difference was found in participants with comparable L1/L2 vocabulary knowledge in L1 (*M* = 1.35, *SE* = 0.17) compared to L2 (*M* = 1.62, *SE* = 0.17), *b* = −0.27, *SE* = 0.19, *t*(70) = −1.45, *p* = .151. In Study 2, the interaction effect between vocabulary balance and language was not significant (unequal vocabulary knowledge: L1 *M* = 1.04, *SE* = 0.16, L2 *M* = 0.65*, SE* = 0.16; equal vocabulary knowledge: L1 *M* = 1.11, *SE* = 0.14, L2 *M* = 1.02*, SE* = 0.14). In Study 3, the main effect of language was significant (*d* = 0.60), indicating lower comprehension on the situation model level in L2 (*M* = 0.55*, SE* = 0.12) compared to L1 (*M* = 0.83, *SE* = 0.12) among participants with unequal L1/L2 vocabulary knowledge. In Study 4, the main effect of language was also significant (L1 *M* = 1.36, *SE* = 0.11, L2 *M* = 1.00*, SE* = 0.11; *d* = 0.65).

## Discussion

In four independent studies, we examined mind-wandering frequency during reading in participants’ first (L1) and foreign language (L2) across different language pairs: L1 German/L2 English (Study 1), L1 French/L2 English (Study 2), L1 English/L2 German (Study 3), and L1 German/L2 French (Study 4). Participants with lower L2 vocabulary knowledge relative to their L1 showed more mind wandering during L2 than L1 reading. Moreover, in Studies 1 and 2, we found no evidence of differences in mind wandering between L1 and L2 reading for participants with comparable L1/L2 vocabulary knowledge. To our knowledge, this study is the first to investigate mind wandering during L2 reading. Our findings align with previous research showing that mind wandering increases with task demands (Feng et al. 2013; Kahmann et al., 2022; Mills et al., 2015; Soemer & Schiefele, 2019). However, the present research contributes by examining individual differences in language proficiency as a source of processing demands, rather than manipulating text difficulty.

Additional measures of comprehension, intentionality of mind wandering, and perceived difficulty provide insight into the mechanisms underlying this effect. In Study 2, L2 reading was associated with more *intentional* mind wandering, whereas in Studies 3 and 4, L2 reading was associated with more *unintentional* mind wandering. One possible explanation is that lower L2 vocabulary knowledge in Studies 3 and 4 impaired comprehension at the textbase and situation model levels. Under these conditions, task demands may have exceeded available executive-control resources, allowing automatic, task-unrelated thoughts to intrude *unintentionally* (Kane & McVay, 2012; McVay & Kane, 2012). This result is also consistent with the situation model view, which proposes that mind wandering increases when readers have difficulties constructing a situation model of the text.

In contrast, comprehension in Studies 1 and 2 was not impaired on the textbase level during L2 reading, and in Study 2, L2 reading was associated with more *intentional* mind wandering. This finding suggests that when at least textbase comprehension remains intact, mind wandering appears to be driven more by metacognitive evaluations of effort. Participants perceived L2 reading as more effortful, reducing the value of continued engagement and promoting *intentional* disengagement, which is consistent with the cost–benefit account (Kurzban et al., 2013; Shenhav et al., 2017). This interpretation is also supported by the mediation results of perceived difficulty. In Studies 1 and 2, lower L2 vocabulary knowledge increased perceived difficulty, which in turn predicted higher mind-wandering frequency. In Studies 3 and 4, no such mediation emerged because task demands likely consumed most available cognitive resources, leaving limited capacity for metacognitive appraisal of difficulty. Notably, negative correlations between mind wandering and situation-model comprehension were observed in all studies, including Studies 1 and 2 (Study 1: *r* = -.19, *p* = .020; Study 2: *r* = -.34, *p* < .001; Study 3: *r* = -.19, *p* = .022; Study 4: *r* = -.31, *p* < .001), which replicates previous work showing that increased mind wandering impairs comprehension (Bonifacci et al., 2023). In Studies 1 and 2, however, reduced comprehension was more likely a consequence of mind wandering than its cause.

## Limitations and conclusion

We address some limitations of the current research and outline directions for future work. First, we used a vocabulary test to assess participants’ language proficiency. Research has shown that vocabulary knowledge correlates with language proficiency, encompassing receptive and productive skills (Jeon & Yamashita, 2014; Miralpeix & Muñoz, 2018). However, a more comprehensive assessment including reading, listening, speaking, and writing would provide a more comprehensive assessment of communicative competence and overall proficiency.

A second limitation concerns the classification of participants into “comparable” versus “unequal” L1/L2 vocabulary knowledge groups based on LexTale scores. Although LexTale provides an efficient measure of vocabulary knowledge within individual languages, the different language-specific versions of the test are not directly comparable. Consequently, the thresholds we applied to distinguish between “comparable” and “unequal” vocabulary knowledge should not be interpreted as precise indicators of balanced proficiency. Our primary aim was to investigate whether readers exhibit higher mind-wandering frequencies in their less proficient L2 compared to their L1. The categorical analyses therefore function mainly as supportive heuristics to illustrate that among participants classified as having “comparable” L1/L2 vocabulary knowledge, we found no evidence of mind-wandering differences between languages.

Third, we assessed comprehension using the recognition-based method developed by Schmalhofer and Glavanov (1986). This approach is well established to differentiate textbase and situation-model comprehension within a single task, which reduces the likelihood that differences between these levels reflect methodological artifacts. Nevertheless, debates are ongoing as to whether recognition tests fully capture comprehension processes. Future research might therefore benefit from complementing recognition-based assessments with additional measures, such as recall tasks or essays, to obtain a more comprehensive evaluation of comprehension (McCarthy et al., 2018).

A practical implication is that texts should match language proficiency to reduce mind wandering. An avenue for future research would be to investigate whether participants with low L2 proficiency exhibit reduced mind wandering when reading simplified texts with more frequent vocabulary and straightforward language. Such findings would offer further evidence that mind wandering diminishes when text difficulty is appropriately matched with reader proficiency.

Overall, across four conceptual replications, we consistently observed more mind wandering during L2 than L1 reading, demonstrating the reliability and generalizability of this effect. The findings suggest that mind wandering is more likely to occur when cognitive resources are insufficient to meet task demands, which is consistent with the executive control failure account, but they also highlight the role of metacognitive evaluations of difficulty when the reading task remains manageable. The present research underscores the importance of considering language proficiency when investigating mind wandering during reading. Future research should build on this insight by examining how language proficiency interacts with other individual factors, such as working memory, as well as with text difficulty, in shaping mind wandering because these factors jointly determine processing demands.

## Data Availability

The experimental texts are available from the authors upon request. The dataset can be found in the Open Science Framework (OSF) repository at: https://osf.io/4tx2s/overview?view_only=ccec5d5b0eae4b9b977be8d1a56b5aa8

## Footnotes

1 Initially, we preregistered data collection from an L1 French population reading in L2 German (https://aspredicted.org/wz7bt9.pdf). However, because of an insufficient number of participants with this language combination, we instead collected data from participants with L1 French and L2 English.

## Code availability

The reproducible R code can be found in the OSF repository at: https://osf.io/4tx2s/overview?view_only=ccec5d5b0eae4b9b977be8d1a56b5aa8

## Declarations

### Open practices statement

The dataset and reproducible R code can be found in the OSF repository: https://osf.io/4tx2s/overview?view_only=ccec5d5b0eae4b9b977be8d1a56b5aa8. The design and analyses from Studies 2–4 were preregistered (https://aspredicted.org/wz7bt9.pdf). We confirm that the studies were registered prior to data collection.
